# The Management of the COVID-19 Pandemic Evidences the Need to Transform Spain’s Public Health Education

**DOI:** 10.3389/ijph.2022.1604907

**Published:** 2022-04-28

**Authors:** Miguel Angel Luque-Fernandez, Aurelio Tobias

**Affiliations:** ^1^ Non-Communicable Disease and Cancer Epidemiology Group, Instituto de Investigación Biosanitaria de Granada (ibs.GRANADA), Granada, Spain; ^2^ Consortium for Biomedical Research in Epidemiology and Public Health (CIBER Epidemiología y Salud Pública, CIBERESP), Madrid, Spain; ^3^ Andalusian School of Public Health, Granada, Spain; ^4^ Inequalities in Cancer Outcomes Network, Department of Non-Communicable Disease Epidemiology, London School of Hygiene and Tropical Medicine, London, United Kingdom; ^5^ Institute of Environmental Assessment and Water Research (IDAEA), Spanish Council for Scientific Research (CSIC), Barcelona, Spain

**Keywords:** Covid, public health education, epidemiology and biostatistics, schools of public health, education impacts

The Lowy Institute, an independent international policy think tank, ranked Spain in the lower quartile measuring the comparative effectiveness of countries’ handling the 2020 COVID-19 pandemic (ranking as of January 9, 2021 was 78 out 98, and of March 13, 2021 was 80 out 102) [1]. Recently, a multi-country study examined excess mortality in 2020 across five European countries showing that excess mortality in 2020 varied widely between countries and within countries. Still, Spain experienced the largest excess mortality among the five countries studied [2]. A robust public health system and organisation might have influenced the response to the pandemic [3]. However, public health and social care services in Spain were not efficient during the first pandemic waves [4, 5].

In the late 1980’s, access to public health education was organised around a medical speciality in preventive medicine and public health. Thus, concentrating efforts on the professional development of medical doctors in public health [6]. Therefore, medicine dominated the essential multidisciplinary and mixed field of public health, academically and professionally. At the same time, other scientific disciplines were excluded from professional access to the field of public health. Consequently, Spain’s public health development and empowerment dynamics were diminished compared to its international peers. Additionally, the politicisation and partisan implementation of public health by regional and national health administrations has resulted in the closure of most of Spain’s public health schools. These institutions were not conceptualised as independent structures linked to the university, where a multidisciplinary curriculum would have been a notable differentiating feature.

The COVID-19 pandemic has shown that Spain urgently needs to implement a profound reform after the evidence of areas where public health and the health and social care system need to be improved [3]. Particularly, public health education and services need reforms. Professional access to public health, epidemiology, and field epidemiology in health services across the country need to be open to other professions and disciplines rather than just medicine. Postgraduate training in epidemiology, biostatistics, and public health requires a reorganisation and transformation. The curriculum is not homogenous, and there is no consensus on how they must be taught [7]. We need university-based public health schools attached to health science campuses. The postgraduate degrees should be identical to those in many other international schools that have organised their structures and educational programmes independently from faculties of medicine and the health administration, like in the United States and the United Kingdom.

However, the forthcoming establishment of a national public health agency will offer an excellent opportunity to develop concomitants educational and professional reforms in Spain’s public health system. Independent professionals and multidisciplinary staff with high proficiency in epidemiology, biostatistics, health data science, public health, health policy, and global public health would be required to improve the capacity to respond to future public health crises at the local and international scales, as revealed by the COVID-19 pandemic.
